# Two-Dimensional Cutting (TDC) Vitrectome: In Vitro Flow Assessment and Prospective Clinical Study Evaluating Core Vitrectomy Efficiency versus Standard Vitrectome

**DOI:** 10.1155/2016/3849316

**Published:** 2016-04-12

**Authors:** Mitrofanis Pavlidis

**Affiliations:** Augencentrum Köln, Josefstraße 14, 51143 Cologne, Germany

## Abstract

*Purpose*. To evaluate comparative aspiration flow performance and also vitrectomy operating time efficiency using a double-cutting open port vitreous cutting system incorporated in a two-dimensional cutting (TDC, DORC International) vitrectome design versus standard vitreous cutter.* Methods*. In vitro investigations compared aspiration flow rates in artificial vitreous humor at varying cutter speeds and vacuum levels using a TDC vitrectome and a standard vitrectome across different aspiration pump systems. A prospective single-centre clinical study evaluated duration of core vitrectomy in 80 patients with macular pucker undergoing 25-gauge or 27-gauge vitrectomy using either a TDC vitrectome at 16,000 cuts per minute (cpm) or standard single-cut vitrectome, combined with a Valve Timing intelligence (VTi) pump system (EVA, DORC International).* Results*. Aspiration flow rates remained constant independent of TDC vitrectome cut rate, while flow rates decreased linearly at higher cutter speeds using a classic single-blade vitrectome. Mean duration of core vitrectomy surgeries using a TDC vitreous cutter system was significantly (*p* < 0.001) shorter than the mean duration of core vitrectomy procedures using a single-cut vitrectome of the same diameter (reduction range, 34%–50%).* Conclusion*. Vitrectomy surgery performed using a TDC vitrectome was faster than core vitrectomy utilizing a standard single-action vitrectome at similar cut speeds.

## 1. Introduction

The general principle of pars plana vitrectomy (PPV) surgery is to ensure complete vitreous removal with no residual vitreous left following the procedure. A principal goal in PPV is to minimize vitreous traction by removing only the target ocular tissue, without inadvertently drawing unwanted tissue into the vitrectomy probe port or creating distant traction that might cause iatrogenic retinal tears or other complications. The degree of retinal traction created by vitrectomy cutters is influenced by the effect of time of aspiration, distance from the retina, and cutting rate. Retinal traction increases with increasing aspiration vacuum and proximity to the retina and decreases with higher cut rates [1].

*Evolution of Vitrectomy Cutter Technology*. Developments in vitrectomy probe technology have accelerated in recent years, designed to improve intraoperative surgical control and allow quick core vitrectomy (bulk vitreous removal) and tractionless controlled vitreous shaving. Alterations in geometrical design and size of vitrectomy probe, together with duty cycle, which is the proportion of time the cutter port is open rather than closed relative to a complete opening and closing surgical cutting cycle, and cutting speed provide additional performance capabilities for more efficient and safer surgery.

Aspiration rate produced by smaller-gauge vitreous cutters is proportionally decreased due to a reduced port size and smaller diameter, explained by Poiseuille's law that flow is proportional to the radius of the tube. This necessitates higher infusion and aspiration pressures to remove vitreous when using smaller-gauge vitrectomes. However, enlarging the port diameter of a vitreous cutter to increase flow becomes less effective as the port becomes larger [2]. Oshima et al. confirmed the feasibility of a 27-gauge instrument system for microincisional vitrectomy surgery (MIVS) in a variety of vitreoretinal procedures, pointing out that such a system might reduce concerns about wound sealing complications in selected cases [3].

The concept of a double-cutting instrument for use in ophthalmic surgery was first patented in 1992 [4]. When vitreous enters the inner aperture, it is cut first in a forward motion and then again during the backward motion. Nearly two decades on, it was suggested that a dual port vitreous cutter system might allow surgeons to perform bulk vitrectomy more efficiently [5]. Rizzo et al. developed a modified vitrectomy probe design with an extra aspiration port in the internal capilair to extend overall duty cycle [6]. The idea involved inclusion of an opening in the internal guillotine pipe or inner vitrectome sleeve. Investigators found that, using modified 23-gauge vitrectomy probes, the time of aspiration remained almost constant irrespective of cutting speed, indicating almost no reduction of flow but, more importantly, that aspiration time was significantly reduced compared with a standard single port cutter.

*Two-Dimensional Cutting (TDC) Vitrectome Design*. The concept of a double-action surgical cutting probe has only recently been developed and incorporated into modern vitrectomy instrumentation probes that feature 2 cutter openings in the guillotine shaft, thereby performing a vitreous cutting action on both forward and backward stroke of the probe device. The principal advantages of this novel guillotine sleeve design included a doubling of cut rate, increased flow, and potentially decreased retinal traction or force exerted by the probe.

In 2013 the author in cooperation with DORC International developed a newer vitrectome design, introducing a modified vitreous cutter technology called two-dimensional cutting (TDC) vitrectome system, launched in conjunction with the EVA ophthalmic surgical platform, a new aspiration system designed to provide both flow and vacuum control mode vitrectomy for enhanced intraoperative fluidics stability.

**Figure 1:**
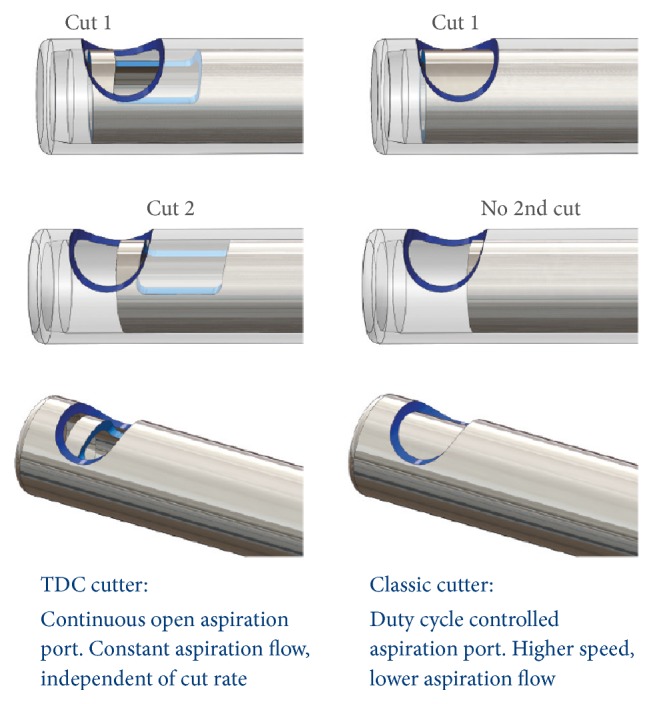
TDC vitrectome design and classic single-cut vitrectome.

The TDC vitrectome comprises a tubular outer part and an axially movable tubular inner part arranged in the outer part [7]. The outer part has a closed distal end and the inner part an open distal end. Both parts have an opening in the tube, allowing continuous aspiration of tissue during a complete cutter cycle movement. The inner tube has been designed with a rectangular aperture for increased and continuous flow functionality. The axial position of the distal cutting edge of the inner part as a function of the circumferential direction initially proceeds towards the proximal end and then back to the distal end again.  Figure 1 illustrates core design elements of a TDC vitrectome and a classic single-cut vitrectome.

The TDC vitrectome performs a double-cutting movement during one complete cycle that is twice the rate of the former simple single-cut vitrectome model, effectively cutting vitreous at up to 16,000 cpm. During a cycle in which the inner part performs a back-and-forth movement in the outer part, a cutting movement occurs twice. A first cutting movement occurs by cooperation of the distal end of the inner part with the distal cutting edge of the outer part. The second cutting movement is realized through cooperation of the distal cutting edge of the inner part with the proximal cutting edge of the outer part. The port diameter of this new TDC vitrectome is larger than previous vitrectome designs (for the 23-gauge TDC vitrectome, e.g., the port is positioned 0.22 mm from the distal end and measures 0.41 mm long and 0.45 mm wide), theoretically allowing a greater amount of tissue to be captured per cutting cycle. Owing to the increased cutting and snipping capacity, the surgical intervention can be shortened and, moreover, the traction exerted on the ocular tissue drawn in during the aspiration phase decreases while the suction flow increases.

We report below the methodologies and findings from in vitro comparisons of aspiration flow dynamics utilizing TDC and standard vitrectomes connected to different surgical platforms, together with methods and results of a prospective surgical case series study evaluating duration of core vitrectomy procedures and therefore comparative flow efficiency performance, using a TDC vitrectome system versus standard vitreous cutter system.

## 2. Methods

### 2.1. In Vitro Aspiration Flow Rate Tests

In vitro evaluations were undertaken to assess volumetric aspiration flow rates (the main outcome measure) of a two-dimensional cutting vitrectome compared with a standard vitrectome of the same gauge. Vitreous cutters, sizes 25-gauge and 27-gauge for both standard blade and TDC vitrectomes, were connected to a DORC EVA ophthalmic surgical unit, and test measurements were obtained for varying vacuum levels and at different cutter speeds up to 8,000 cpm. A standard blade vitrectome of both gauges was also evaluated for aspiration flow rate at varying cut rates when connected to an Associate 6000 machine. The Associate system integrates a peristaltic and Venturi pump system, while the EVA phaco/vitrectomy system incorporates a VacuFlow Valve Timing intelligence (VTi) fluidics control system, which provides instantaneous flow or vacuum mode aspiration.

A cut rate and a vacuum level were set on a EVA machine according to the following range of tested settings: cut rate of 0 cpm, 1,000 cpm, 2,000 cpm, 3,000 cpm, 4,000 cpm, 5,000 cpm, 6,000 cpm, 7,000 cpm, and 8,000 cpm; and a vacuum level of 350 to 550 mmHg. The highest vacuum limit was set to 550 mmHg due to the stability of the vacuum til 550 mmHg. Above 550 mmHg every pump system delivers fluctuating vacuum parameters, which are not appropriate for exact evaluations.

Before each test, a priming procedure was performed to ensure that the aspiration tubing of the cutter was completely filled with water and that the cutter was positioned with its tip into a cup filled with fluid. The cup was placed on a high precision balance (0.01 g) which was connected to a computer. After activation of the vitreous cutter, a small time was allowed for attainment of a constant vacuum level in the aspiration tubing. When the vacuum level was constant, the weight reduction of the fluid in the cup was measured and the aspiration flow calculated by dividing the weight reduction by the time elapsed.

Initially, these tests were performed with water. The same tests were then performed using artificial vitreous humor as aspirating fluid. The artificial vitreous, consisting of a mixture of deionized water, agar, and hyaluronic acid sodium salt, was produced according to a protocol published by Kummer et al. [8] and used as a suitable dummy tissue to represent human vitreous humor for these in vitro experiments. For all tests performed, a density of 1.0 kg/L was used to covert the measured mass flow to a volume flow. Using artificial vitreous, 3 trials of 30 seconds' duration were performed. Per gauge size (and type) at least 2 vitreous cutters were tested. The average aspiration flow was calculated by averaging the measurement results of the different trials and the different vitreous cutters of the same type and gauge.

### 2.2. In Vivo Comparative Efficiency Performance

We evaluated overall vitrectomy cutting time (in seconds) required to remove core vitreous in 80 patients diagnosed with macular pucker as part of a comparative evaluation of flow and operating duration using a TDC vitrectome and former standard single-cut vitrectome design (25-gauge and 27-gauge systems, DORC) during core vitrectomy procedures.

Caucasian subjects aged over 50 years, diagnosed with macular pucker, refraction of +2 to −2 diopters, no previous intraocular surgery, and having a detached posterior vitreous were recruited. Inclusion criteria were chosen to ensure adherence to a similarity principle, with similar vitreous liquefaction, similar case duration and difficulty, similar vitreous volume, and similar overall ocular conditions having had no prior ocular surgical intervention. Patients with a diagnosis at baseline of glaucoma, asteroid hyalosis, acute or chronic uveitis, or trauma were excluded. A total of 80 patients were enrolled, with equal numbers, or 20 eyes, randomly allocated to one of four surgical treatment groups: 25-gauge standard vitreous cutter PPV, 25-gauge TDC vitreous cutter PPV, 27-gauge standard vitreous cutter PPV, and 27-gauge TDC vitreous cutter PPV; all procedures performed using the EVA surgical system.

Core vitreous volume was defined intraoperatively as the central area of the vitreous that could be visualized via a 90-diopter (D) PPV lens using the EIBOS I (Haag-Streit) viewing system. During central vitrectomy in the visible area of the 90 D lens, the eye was left stable so as to standardize the viewing field of vitreous removal and to allow for more precise evaluation. The field of the 90 D EIBOS lens remains constant and independent of distance to the eye. Using the EVA vitrectomy surgery machine with identical configuration settings and machine parameters for core vitrectomy mode, aspiration power was set at a maximum 680 mmHg, while vitrectome cutting speed used throughout the vitrectomy procedure was as high as 8,000 cuts per minute, equivalent to 16,000 cpm, using a TDC vitrectome.

All vitrectomized subjects were adequately informed prior to surgery about standard PPV for epiretinal membrane and core vitrectomy measurements and signed a consent form. Time measurement of core vitrectomy duration was made by a secondary person without a need to change standard vitrectomy procedures of PPV for epiretinal membrane removal. The study adhered to the tenets of the Declaration of Helsinki, and local regulatory requirements were fulfilled. Time and flow data were analyzed using linear least squares regression analyses and two-tailed *t*-tests. Performance of the new generation 25-gauge and 27-gauge TDC vitrectomes was analyzed relative to the current generation or standard 25-gauge and 27-gauge cutter. Statistical significance (*p* value) of comparison between PPV durations performed using standard and TDC vitreous cutters was set at 0.001.

## 3. Results

### 3.1. In Vitro Assessment of Aspiration Flow Rates

**Figure 2:**
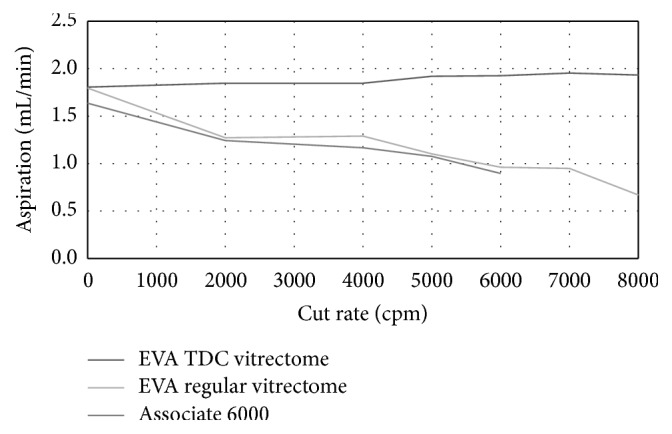
Aspiration flow rates of 25G TDC and standard vitrectomes @ 350–550 mmHg.

**Figure 3:**
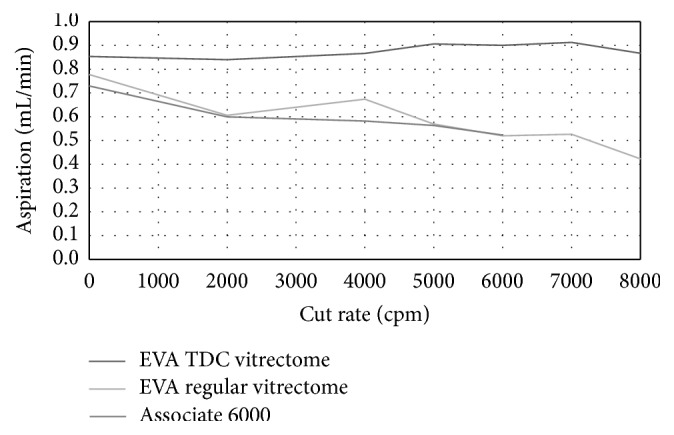
Aspiration flow rates of 27G TDC and standard vitrectomes @ 350–550 mmHg.

For in vitro investigations conducted in artificial vitreous, as the cutting rate increased from 0 to 8,000 cpm using a TDC vitrectome connected to EVA surgical aspiration system, aspiration flow rate remained constant, while aspiration flow rate steadily decreased at higher cut speeds with a standard vitrectome connected to the EVA or Associate surgical systems. Figures 2 and 3 show illustrative aspiration curve analyses obtained from comparative test evaluation measurements using a 25-gauge and 27-gauge TDC vitreous cutter and a classic 25-gauge and 27-gauge cutter in combination with the EVA machine and Associate platform at varying cutting rates, measured at a vacuum setting of 350 to 550 mmHg.

Table 1 includes measured aspiration flow rates obtained with the TDC and standard single-cut vitrectomes (25-gauge and 27-gauge) combined with EVA for varying vacuum levels and at cut speeds up to 8,000 cpm, confirming continuous high aspiration flow rates using a TDC vitrectome that are maintained constant at cut rates from 0 cpm to 8,000 cpm.

At a vacuum set at 350 mmHg, average aspiration flow rate measured using a 25-gauge TDC vitrectome in combination with the EVA surgical system ranged from 1.8 mL/min to 2.0 mL/min at cut rates between 0 cpm and 8,000 cpm; for the classic vitrectome connected to the EVA system, the flow rate measurement declined to 0.7 mL/min at 8,000 cpm from a flow rate of 1.3 mL/min at 2,000 cpm. When the classic 25-gauge vitrectome was connected to the Associate 6000 system, aspiration flow rate decreased from 1.6 mL/min at 0 cpm to 0.9 mL/min at 6,000 cpm cut rate, with vacuum set at 350 to 550 mmHg. Aspiration flow rate with a 27-gauge TDC vitrectome in combination with the EVA surgical system at maximum mmHg ranged between 2.0 mL/min and 2.1 mL/min and between 2,000 cpm and 8,000 cpm cut rates, compared with a linear decrease in measured aspiration flow rate at increasing cutter speeds with the 27-gauge classic vitrectome connected to the EVA system.

### 3.2. In Vivo Evaluations of Surgical Efficiency in Core Vitrectomy

In the comparative case series clinical study, the mean duration of core vitrectomy procedures using 25-gauge and 27-gauge TDC vitreous cutter system was statistically significantly (*p* < 0.001) shorter than the mean operating duration for core PPV performed utilizing a standard single-cut vitrectome of the same gauge.

Table 2 tabulates the mean duration of core vitrectomy procedures for each surgical intervention group assessed. The data show that the mean duration of PPV performed using a 27-gauge TDC vitrectome was 34% shorter (−83.0 seconds) than the mean duration measured for PPV using a standard 27-gauge single-cut vitrectome; similarly, for 25-gauge PPV, the respective reduction in duration of surgery using a TDC vitrectome system compared with a standard vitrectome was 50% (−74.39 seconds).

## 4. Discussion

In vitro tests demonstrate that a more predictable and consistent flow of vitreous around the instrument probe is achieved using a TDC vitrectome compared with a regular vitrectome system. A TDC vitrectome delivered good overall stability in aspiration flow rate that is independent of cut speed. At a cut rate of 8,000 cpm, equivalent to 16,000 cpm, we postulate that a TDC vitrectome, by cutting the vitreous into smaller bites, may induce less iatrogenic breaks. Moreover, the continuous open port of the TDC vitreous cutter permits greater tissue removal efficiency that is unaffected by cutter velocities, showing the potential of TDC vitrectome technology for faster, less turbulent, and potentially safer smaller-gauge vitrectomy.

The reported comparative evaluation of core vitrectomy duration in 80 patients undergoing surgery for macular pucker (epiretinal membranes) revealed that surgical case time using TDC vitrectome PPV is less than vitrectomy operating time performed using a standard single port cutter of the same gauge. Vitrectomy surgery using a TDC vitrectome resulted in faster core vitrectomy, a finding that was consistent across both 25-gauge and 27-gauge instrumentation surgery groups.

Fluidic stability and control during vitrectomy is essential. Retinal surgeons choose high vitreous cutter rates so as to maximize fluidic stability and reduce unwanted force or traction. Higher cutting rates using a TDC vitrectome in combination with continuous uninterrupted aspiration flow as a result of 2 open cutting ports help to ensure faster complete vitreous removal. If the higher double-cutting rate minimizes unwanted vitreous traction and reduces the risk of iatrogenic retinal damage is object of a safety designed ongoing study. Early 25-gauge vitrectomy systems were marked by reduced fluid flow and longer vitrectomy duration compared with 20-gauge systems [9]. However, the design of new generation dual-opening vitreous cutters effectively overcomes these initial limitations by providing for consistent flow irrespective of the cut rate used during vitrectomy surgery.

Findings from this small comparative case series assessment are supportive of the efficiency of TDC vitrectome technology and of faster cut speeds for vitrectomy surgery. Results suggest significantly decreased operating time for core vitrectomy. The author's experience performing microincisional vitrectomy with small-gauge instruments suggests that the approach is particularly well suited for microsurgical maneuvers that may be required for proliferative diabetic retinopathy, vitreomacular traction, macular hole closure, and high myopia cases. Evaluations reported herein are nonetheless limited by small research scale and by the fact that the same surgeon performed all vitrectomies in this single-centre clinical assessment. There is undoubtedly a surgical learning curve involved in mastering the technique of using small-gauge vitrectomy instruments, typically involving the first 20 or so cases. Future follow-up studies might usefully evaluate postoperative visual, anatomic, and safety outcomes.

Survey trends illustrate growing utilization of sutureless microincision vitrectomy in everyday retina practice [10]. Advocates of microincisional vitrectomy instrumentation highlight surgical advantages compared with conventional 20-gauge surgery in addition to sutureless vitrectomy capability, namely, reduced operating time, greater precision in performing delicate maneuvers, less tissue manipulation, and reduced postoperative inflammation and rapid visual recovery [11, 12]. A report by the American Academy of Ophthalmology in 2010 noted that, compared with 20-gauge vitrectomy, small-gauge vitrectomy is associated with significantly lower levels of patient discomfort and ocular inflammation, with faster improvement in visual acuity, and an acceptably low incidence of adverse events comparable to those observed for 20-gauge vitrectomy [13].

Overall, vitrectomy case duration using a TDC vitrectome in combination with the EVA surgical machine was shorter than vitrectomy operating time using a standard or classic single-cut vitrectome in patients undergoing vitrectomy for epiretinal membranes. Faster operating times offer the potential of reduced costs as well quicker postoperative rehabilitation [14]. Reported findings suggest that a TDC vitrectomy probe provides greater operating efficiency than conventional vitreous cutter instrumentation during sutureless small-gauge vitrectomy.

## Figures and Tables

**Table 1:** Comparison of aspiration flow rates (mL/min) using TDC and standard single-cut vitrectomes connected to the EVA surgical system at varying cut rates and different vacuum pressures, in vitro tests performed using artificial vitreous humor as the aspirating fluid.

Cut rate (cpm)	0	2,000	4,000	5,000	6,000	7,000	8,000
25G TDC vitrectome flow rate (mL/min)							
350 mmHg	1.8	1.8	1.8	1.9	1.9	2.0	1.9
550 mmHg	3.2	3.3	3.2	3.3	3.3	3.3	3.3
Max mmHg	4.3	4.3	4.2	4.3	4.2	4.2	4.2
25G standard vitrectome flow rate (mL/min)							
350 mmHg	1.8	1.3	1.3	1.1	1.0	0.9	0.7
550 mmHg	3.1	2.2	2.2	1.8	1.6	1.6	1.1
Max mmHg	3.9	2.8	2.7	2.3	2.0	1.9	1.3
27G TDC vitrectome flow rate (mL/min)							
350 mmHg	0.9	0.8	0.9	0.9	0.9	0.9	0.9
550 mmHg	1.6	1.6	1.6	1.6	1.6	1.6	1.6
Max mmHg	1.9	2.0	2.0	2.1	2.1	2.1	2.0
27G standard vitrectome flow rate (mL/min)							
350 mmHg	0.8	0.6	0.7	0.6	0.5	0.5	0.4
550 mmHg	1.5	1.1	1.1	0.9	0.8	0.8	0.6
Max mmHg	1.9	1.4	1.4	1.2	1.0	1.0	0.7

TDC: two-dimensional cutting; cpm: cuts per minute; mmHg: millimeters of mercury.

**Table 2:** Mean duration of core vitrectomy operating time for 27-gauge and 25-gauge surgeries utilizing either a standard single-cut or a TDC vitrectome.

Parameter	25-gauge vitrectomy		27-gauge vitrectomy	
	Standard vitrectome (*n* = 20)	TDC vitrectome (*n* = 20)	Standard vitrectome (*n* = 20)	TDC vitrectome (*n* = 20)
Mean core vitrectomy duration (SD), in seconds	148.19 (25.14)	73.80 (18.61)	242.71 (25.52)	159.71 (29.47)
*p* value: significance of comparison between core vitrectomy durations performed using a standard and a two-dimensional cutting (TDC) vitrectome (*t*-test)	*p* < 0.001		*p* < 0.001	

Mean duration of core vitrectomy operating time for 27-gauge and 25-gauge surgeries utilizing either a standard single-cut or a TDC vitrectome is shown above, demonstrating significantly shorter case duration for TDC vitrectomy surgery groups (*n* = 80, 20 eyes assigned, resp., to each surgery group).

## Additional Points

Experimental assessments show maintenance of constant high aspiration flow independent of cutter speed using a two-dimensional cutting (TDC) vitrectome for vitreous removal. A prospective single-centre clinical study assessed duration of core vitrectomy procedure using a TDC vitrectome versus a standard single-cut guillotine vitrectome; the results reveal shorter operating times using a double-cutting TDC vitrectomy probe.
